# The Nogo-B-PirB Axis Controls Macrophage-Mediated Vascular Remodeling

**DOI:** 10.1371/journal.pone.0081019

**Published:** 2013-11-20

**Authors:** Yuka Kondo, Caroline C. Jadlowiec, Akihito Muto, Tai Yi, Clinton Protack, Michael J. Collins, George Tellides, William C. Sessa, Alan Dardik

**Affiliations:** 1 The Vascular Biology and Therapeutics Program and the Department of Surgery, Yale University School of Medicine, New Haven, Connecticut, United States of America; 2 Mie University Graduate School of Medicine, Department of Thoracic and Cardiovascular Surgery, Tsu, Japan; 3 Department of Surgery, University of Connecticut, Farmington, CT, United States of America; 4 Veterans Affairs Connecticut Healthcare Systems, West Haven, Connecticut, United States of America; University of Louisville, United States of America

## Abstract

**Objective:**

Nogo-B mediates vascular protection and facilitates monocyte- and macrophage-dependent vascular remodeling. PirB is an alternate receptor for Nogo-B, but a role for the Nogo-PirB axis within the vascular system has not been previously reported. We examined whether Nogo-B or PirB play a role in regulating macrophage-mediated vascular remodeling and hypothesized that endothelial Nogo-B regulates vein graft macrophage infiltration via its alternate receptor PirB.

**Methods:**

Vein grafts were performed using Nogo and PirB wild type and knockout mice. Human vein grafts were similarly analyzed. The hindlimb ischemia model was performed in PirB wild type and knockout mice. Accompanying in vitro work included isolation of macrophages from PirB wild type and knockout mice.

**Results:**

Increased Nogo-B and PirB mRNA transcripts and protein expression were observed within mouse and human vein grafts. Both Nogo knockout and PirB knockout vein grafts showed increased wall thickness and increased numbers of F4/80-positive macrophages. Macrophages derived from PirB knockout mice had increased adhesion to fibronectin, increased EC-specific binding, and increased numbers of mRNA transcripts of M2 markers as well as MMP3 and MMP9. PirB knockout vein grafts had increased active MMP9 compared to wild type vein grafts. PirB knockout mice had increased recovery from hindlimb ischemia and increased macrophage infiltration compared to wild type mice.

**Conclusions:**

Vein graft adaptation shows increased expression of both Nogo-B and PirB. Loss of PirB, or its endothelial ligand Nogo-B, results in increased inflammatory cell infiltration and vein graft wall thickening. These findings suggest that PirB regulates macrophage activity in vein grafts and that Nogo-B in the vein graft limits macrophage infiltration and vein graft thickening. PirB may play a more general role in regulating macrophage responses to vascular injury. Macrophage inhibition via Nogo-PirB interactions may be an important mechanism regulating vein graft adaptation to the arterial circulation.

## Introduction

Vein graft adaptation after surgical placement of a vein into the arterial circulation results in complex structural changes. While some venous wall thickening appears to be a physiological adaptation to the arterial environment, the mechanisms that regulate this process remain unclear. Outstanding questions remain regarding how wall thickness is controlled and, more fundamentally, how much thickness is needed for successful vein graft adaptation. As such, the differences between normal and aberrant venous remodeling remain poorly understood [1].

Nogo-B, a member of the reticulon protein family, is highly expressed in the vascular system and appears to play a vasculoprotective role. Deficiency of Nogo-B is associated with increased intimal hyperplasia and addition of exogenous Nogo-B can rescue the deficiency [2]–[4]. We have previously described, using a rabbit model of balloon injury, that Nogo-B is a marker of arterial neointimal hyperplasia, but is not involved with arterial adaptive remodeling after injury [3]. In that report, we also showed colocalization between Nogo-B and adventitial macrophages, but we did not appreciate the significance of that finding at that time [3]. We have also previously reported that Nogo-B expression increases during vein graft adaptation, both in the neointima and the adventitia of human vein grafts, as well as in a rat model of vein graft adaptation [5]. Again, in that report, we did not appreciate the significance of this finding, especially the significance of the increase in Nogo-B during venous remodeling compared to the decrease found after arterial injury [3], [5]. Recent work has likewise suggested a connection between Nogo-B and control of macrophage activity [6], [7]. It has been suggested that Nogo-B plays a role in regulating macrophage-mediated arterial remodeling, although this finding has not been confirmed in venous adaptation [6].

Traditional receptors for Nogo-B include both the Nogo receptor (NgR) and the Nogo-B receptor (NgBR) [8]. More recently, Paired Immunoglobulin-Like Receptor B (PirB), an inhibitory regulatory receptor found on macrophages and inflammatory cells, has also been identified as a potential binding site for loop of Nogo-B. Although the importance of Nogo-PirB interactions has been shown within the central nervous system, similar activity within the vascular system has not yet been reported [9]–[11].

Our data suggests that PirB may play a role in regulating macrophage-mediated vascular remodeling, both during venous as well as during arterial remodeling. We hypothesized that during vein graft adaptation, endothelial Nogo-B serves as a ligand for PirB on circulating cells such as monocytes, thereby regulating macrophage trafficking within vein grafts.

## Methods

### Antibodies and Reagents

Primary antibodies to the following antigens and reagents were obtained as follows: Nogo (N-18), HA-probe (Santa Cruz Biotechnology); F4/80, alpha smooth muscle actin, CD68 and CD31 (Abcam); PirB (LILRB3; Millipore AB2269), Nogo-B (R&D); murine macrophage colony-stimulating factor (PeproTech); histopaque 1077 (Sigma-Aldrich); TRIzol Reagent (Invitrogen); RNeasy Mini kit (Quiagen); SuperScript III First-Strand Synthesis Supermix (Invitrogen); Fibronectin Cell Adhesion Assay (ScienCell Research Laboratories).

### Human Vein Grafts

Human veins, arteries, and patent vein grafts were obtained as previously described and as detailed in the **Ethics Statement** below [5].

### Vein Graft Model

All animal procedures were approved as detailed in the **Ethics Statement** below. Infrarenal abdominal aortic vein grafts were created in adult (12–15 weeks old) C57BL/6, Nogo-A/B wild type (WT), PirB WT, and PirB knockout (KO) mice as previously described using thoracic vena cava from donor C57BL/6, Nogo-A/B WT, Nogo-A/B KO, and PirB WT mice (**Table 1**) [12]–[14]. PirB WT and KO mice were graciously obtained from Dr. Carla Shatz (Stanford, CA). Vein grafts were followed postoperatively using ultrasound imaging for patency and wall thickness. Computer densitometry was used to measure intima-media area as well as total vein graft area.

**Table 1:** Mouse vein graft configurations.

Vein Graft Configuration	Host	Vein Donor
WT	C57BL/6	C57BL/6
Nogo WT	Nogo-A/B^+/+^	Nogo-A/B^+/+^
Nogo KO	Nogo-A/B^+/+^	Nogo-A/B^−/−^
PirB WT	PirB^+/+^	PirB^+/+^
PirB KO	PirB^−/−^	PirB^+/+^

### Hind-Limb Ischemia Model

All animal procedures were approved as detailed in the **Ethics Statement** below. The femoral, profunda, and epigastric arteries were isolated, doubly ligated and transected in 15 week old PirB WT and PirB KO mice. The superficial femoral artery caudal to the major branch points was then dissected, ligated and excised in its entirety. Care was taken to ensure preservation of the femoral vein and nerve. Flow was measured at baseline, after induction of ischemia, and weekly using invasive Doppler as previously described [15].

### Primary Mouse Lung Endothelial Cell Isolation

Mouse lung endothelial cells (EC) were isolated as previously described. Briefly, under sterile conditions, lung tissue was collected from 4 week old mice following euthanasia. Tissue was minced, treated with Type I collagenase, and incubated with magnetic beads containing anti-mouse CD31. A magnetic separator was used to isolate beads with adherent CD31-positive cells. Cells were then incubated and allowed to reach confluency, after which a second sort was performed. CD31 FACS analysis was used to confirm final culture purity.

### Macrophage Isolation

Under sterile conditions, bone marrow was collected from the long bones of PirB WT and PirB KO mice. Mononuclear cells were isolated by gradient centrifugation and macrophages were selectively cultured using macrophage colony-stimulating factor (20 ng/ml). Macrophages were allowed to achieve confluency and were collected or prepared for experiments on day 8. FACS analysis with F4/80 antibody was performed to ensure cell culture purity.

### Immunohistochemistry

Peroxidase blocking was performed in tissue sections followed by incubation with primary antibodies. Immunohistochemical detection was achieved using DAB substrate. Sections were counterstained using hematoxylin.

### Immunofluorescence

Tissue sections were incubated using primary antibodies followed by incubation with appropriate fluorophore-conjugated secondary antibodies. Secondary detection was performed using Alexa Fluor 488 and 568 and counterstained with DAPI. Images were captured with an AxioImager A1 (Carl Zeiss, Inc.) under identical conditions.

### Quantitative PCR Analysis

Total RNA was isolated from either tissue or cell culture, purified, and digested with DNase-I. Total RNA concentration was measure by spectrophotometer. Reverse transcriptase was used for cDNA synthesis. Amplification specificity was confirmed using 2% agarose gel electrophoresis. Primer efficiencies were determined by melting curve analysis. All data was normalized by GAPDH or 18S amplification.

### Western Blot Analysis

Protein extraction was performed using lysis buffer with protease and phosphatase inhibitors. For animal and human specimens, tissue was snap frozen in liquid nitrogen prior to undergoing protein extraction. Immunoprecipitation samples were incubated overnight with agarose-G beads and either anti-HA-probe or control rabbit IgG antibodies. Standard SDS-PAGE gel electrophoresis was used to analyze sample protein. Densitometry was performed using Image-J software (NIH).

### Fibronectin Adhesion Assay

The assay was carried out following the manufacturer's protocol. In brief, PirB macrophages where isolated as described above and plated at 3×10^4^ density onto either pre-coated BSA or fibronectin wells. Macrophages were incubated for 30 minutes at 37°C and the plate was read at OD_595nm_.

### Macrophage-EC Adhesion Assay

PirB macrophages were isolated as described above. Macrophages were labeled with an immunofluorescent tag using Calein-AM. EC were plated at equal numbers and starved in serum-free media for 6 hours. The media was then removed and PirB WT and KO macrophages were activated with TNF-α and then applied in equal numbers to plated EC. Macrophages were allowed to incubate for 1 hour after which time media and non-adherent macrophages were removed and EC were washed with PBS. Cell counts of remaining adhered macrophages were then performed.

### VEGF-A ELISA Assay

Macrophages derived from PirB WT and KO mice were plated at equal cell densities and cell culture supernatants were collected following a 24-hour period of incubation. Analysis of the supernatants was then performed using a mouse VEGF-A ELISA assay (R&D Systems, Minneapolis, MN). Samples were analyzed in duplicate form and assays were used as directed by the manufacturer's product protocol.

### Statistical Analysis

Results are expressed as the mean ± standard error of the mean (SEM). Comparisons between groups were performed using either t-test or analysis of variance (ANOVA) as appropriate and indicated in the legends. P values less than 0.05 were considered statistically significant (Prism 6; GraphPad software, La Jolla, CA).

### Ethics statement

Human specimens were obtained with the approval of the Yale University Human Investigation Committee, Protocol 9908011041. Organ donors had informed signed consent by their family for donation that included use of tissues for research; the Human Investigation Committee waived additional consent for this study. This study was carried out in strict accordance with the recommendations in the Guide for the Care and Use of Laboratory Animals of the National Institutes of Health. The protocol was approved by the Institutional Animal Care and Use Committee of Yale University (protocol 2010-10896). All surgery was performed under anesthesia, and all efforts were made to minimize suffering.

## Results

### Increased expression of both Nogo-B and PirB in vein grafts

**Figure 1:**
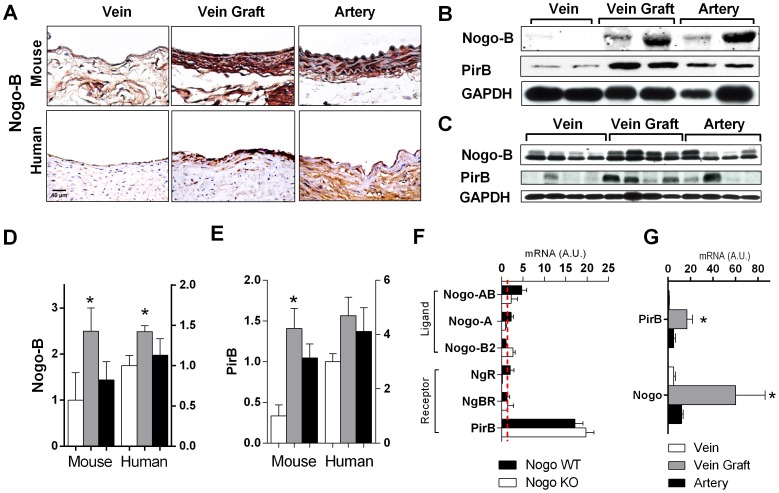
Increased Nogo-B and PirB expression in mouse and human vein grafts. (**A**) Representative immunohistochemical staining images in mouse (top row) and human (bottom row) vein (left), vein graft (center), and aorta (right). n = 4. Scale bar, 40 µm. (**B**) Representative Western blot analysis showing Nogo-B and PirB up-regulation in individual mouse vein grafts; n = 2 matched specimens are shown of n = 4 samples. (**C**) Representative Western blot analysis showing Nogo-B and PirB up-regulation in individual human vein grafts; n = 4 unmatched specimens are shown. (**D**) Bar graph shows densitometry of Nogo-B bands shown in Panels (B) and (C) during mouse and human vein graft adaptation. *, p<0.05; t-test (vein vs. vein graft). (**E**) Bar graph shows densitometry of PirB bands shown in Panels (B) and (C) during mouse and human vein graft adaptation. *, p<0.05; t-test (vein vs. vein graft). (**F**) Bar graph shows summary of gene expression during mouse vein graft adaptation; the number of mRNA transcripts expressed during vein graft adaptation is compared to the number expressed in the wild type pre-implantation inferior vena cava (IVC), denoted by the red broken line. n = 4. ▪, Nogo WT; □, Nogo KO vein grafts. (**G**) Bar graph shows Nogo-B and PirB mRNA transcripts during human vein graft adaptation. Analysis of n = 8 veins, 6 vein grafts, and 5 arterial human specimens. *, p<0.05; t-test (vein vs. vein graft).

To determine a role for Nogo-B and PirB during vein graft adaptation, the expression of Nogo-B and PirB was examined in both patent human vein grafts as well as in a mouse model of vein graft adaptation. There was less Nogo-B immunoreactivity in veins compared to arteries, although the specificity of Nogo-B immunoreactivity in the arteries may reflect species-differences between humans and mice (**Figure 1A**). However, compared to lower levels of Nogo-B in control veins, increased Nogo-B immunoreactivity was observed in both mouse and human vein grafts (**Figure 1A**). Western blot confirmed increased levels of Nogo-B protein in both mouse vein grafts (**Figure 1B**) and human vein grafts (**Figure 1C**) compared to control veins. Similarly, there was increased expression of PirB, a receptor for Nogo-B, in both mouse vein grafts (**Figure 1B**) and human vein grafts (**Figure 1C**) compared to control veins.

Quantification of Western blot densitometry showed increased levels of Nogo-B (p = 0.0381, n = 4) and PirB (p = 0.0130, n = 6) protein in mouse vein grafts compared to control veins (**Figures 1D, 1E**). Similarly, analysis of human vein graft densitometry showed increased Nogo-B protein (p = 0.0277, n = 4) and a trend towards increased PirB protein (p = 0.0625, n = 4) compared to control veins (**Figures 1D, 1E**).

mRNA expression patterns for known Nogo ligands and receptors were performed using qPCR analysis in Nogo WT and Nogo KO mouse vein grafts (**Table 1**). Quantification of mRNA levels showed detectable levels of Nogo AB, but not the A or B2 isoforms, in Nogo WT vein grafts; these mRNA levels were reduced below baseline in Nogo KO vein grafts (**Figure 1F**). Expression of mRNA was not detectable for either the Nogo receptor or the Nogo-B receptor, but mRNA for PirB was noticeably higher than baseline both in Nogo WT and KO vein grafts (**Figure 1F**). Similar expression patterns were observed in human vein grafts, with increased mRNA transcripts detected for the ligand Nogo-B (p = 0.0635, n = 4) and its receptor PirB (p = 0.0172, n = 4) (**Figure 1G**). These results are consistent with expression of both Nogo-B, and its receptor PirB, during both human and mouse vein graft adaptation.

### PirB is expressed largely in vein graft macrophages

**Figure 2:**
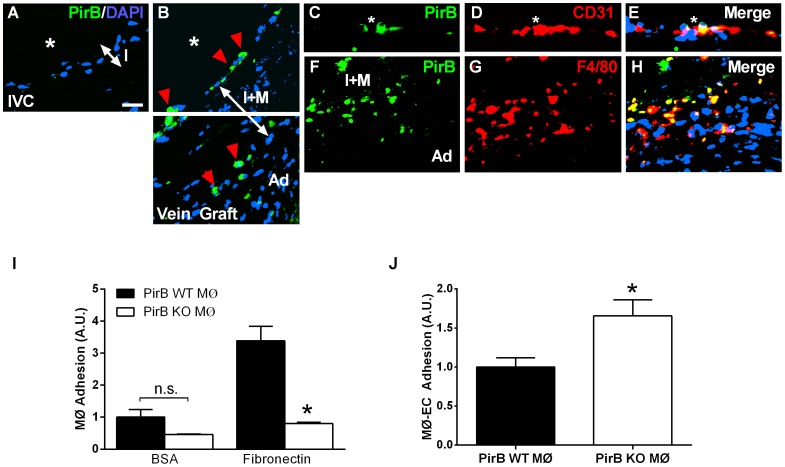
PirB mediates adhesion in vitro and in vivo. (**A**) Representative immunofluorescence image showing lack of PirB signal in the pre-implantation vein. IVC, inferior vena cava. Scale bar, 20 µm. (**B**) PirB positive cells localized to the luminal surface and the interface between the medial and adventitial layers. *, vessel lumen; red arrowhead, PirB positive cells; I, intimal layer; I+M, intima-medial layer; Ad, adventitial layer. (**C–E**) PirB-positive cells on the vein graft luminal vessel surface (**C**) did not co-localize with CD31-positive endothelial cells (**E**). *, vessel lumen; white arrows, PirB-positive cells. (**F–H**) PirB-positive cells in between the medial and adventitial layers (**F**) co-localize with F4/80-positive cells (**G,H**). I+M, intima-medial layer; Ad, adventitial layer. n = 4. (**I**) Bar graph shows macrophage adhesion to bovine serum albumin (BSA) or fibronectin. Macrophages were derived from PirB WT (▪) or PirB KO (□) mice. n.s., not significant. n = 8. *, p<0.0001; ANOVA; post-hoc testing p<0.05. (**J**) Bar graph shows macrophage adhesion to endothelial cells. Macrophages were derived from PirB WT (▪) or PirB KO (□) mice and activated with TNF-α. *, p = 0.0499, t-test; n = 3.

Since both Nogo-B and PirB have increased expression during vein graft adaptation, we next examined whether they colocalized in vivo. We have previously shown that Nogo-B is strongly expressed in the vein graft neointima with much smaller amounts in the vein graft media [5]. Immunofluorescence staining for PirB showed that PirB was not detected in control mouse veins (**Figure 2A**). However, PirB immunoreactivity was detected in both the intima and adventitia of mouse vein grafts (**Figure 2B**). Although PirB was easily detectable at the vein graft CD31-positive intima, there was little colocalization of PirB and CD31 (**Figures 2C–E**), suggesting that PirB-positive cells in vein grafts are not endothelial cells. However, colocalization was observed between PirB and F4/80-positive cells within the vein graft adventitia, suggesting that PirB is present in F4/80-positive macrophages that infiltrate the vein graft (**Figures 2F–H**). These infiltrating cells were also immunoreactive for CD68 (data not shown), consistent with their being macrophages.

Since PirB colocalizes with macrophages during vein graft adaptation, we determined whether PirB could regulate macrophage function in vitro. Loss of PirB resulted in decreased macrophage adhesion to fibronectin (n = 8; p<0.0001) (**Figure 2I**) and increased specific binding, after TNF-α activation, to endothelial cells (n = 3, p = 0.0499) (**Figure 2J**). These results are consistent with PirB playing an inhibitory role in macrophage functions, as previously described [16]–[18]. In addition, a similar phenotype has been reported in Nogo-receptor deficient macrophages [19].

### Nogo-B limits macrophage infiltration and vein graft thickening

Since our data shows that Nogo-B, and its receptor PirB, are both upregulated in vein grafts (**Figure 1**), and that PirB may regulate macrophage homing or adhesion to vein grafts (**Figure 2**), we hypothesized that Nogo-B has a functional role during vein graft adaptation, and that this role might involve macrophages. To test this hypothesis we used a mouse model of vein graft adaptation to compare vein graft adaptation in veins derived from wild type (WT) and Nogo KO mice.

**Figure 3:**
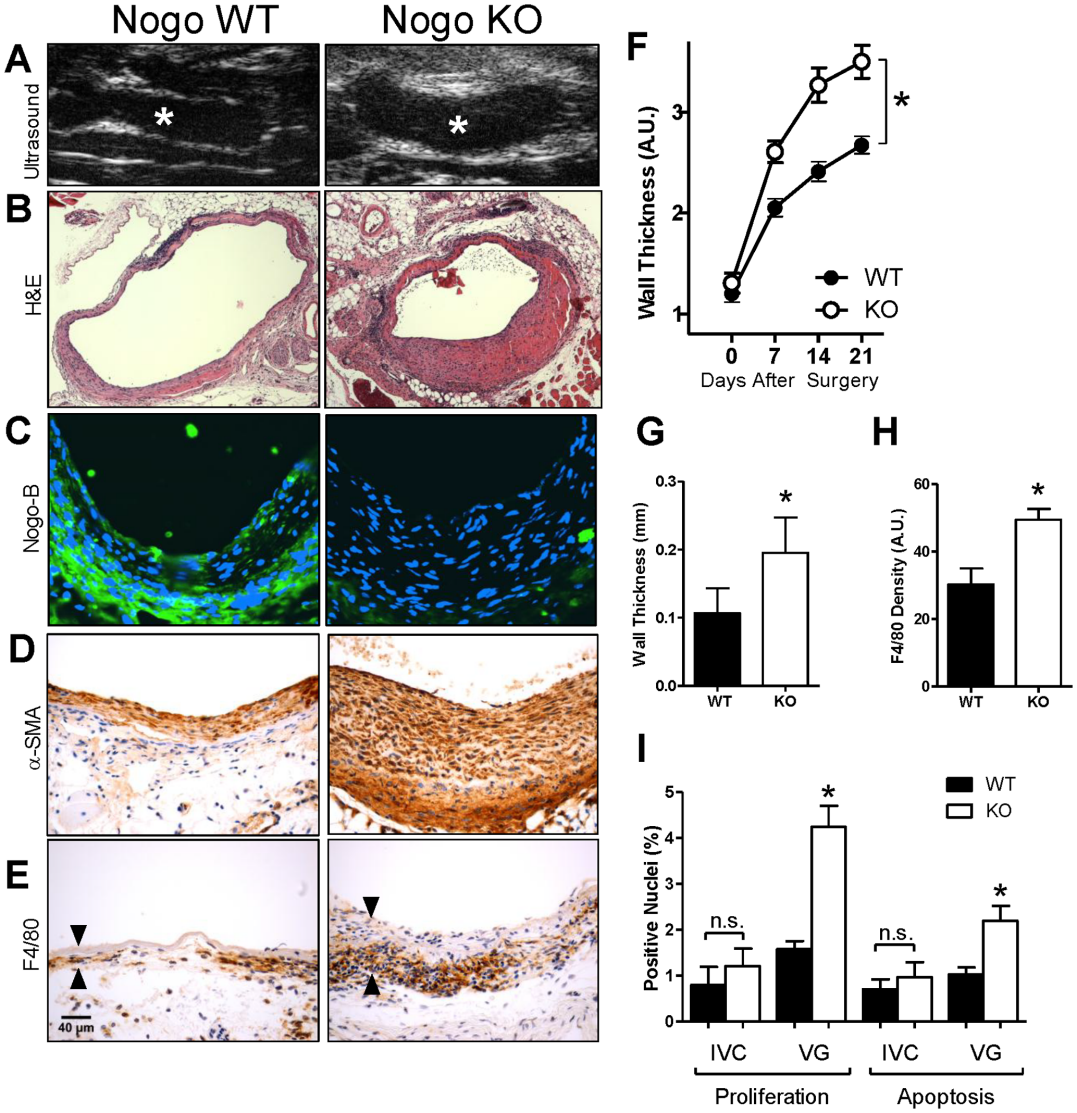
Nogo-B limits F4/80-positive cell infiltration during vein graft adaptation. (**A**) Representative ultrasound images of mouse vein grafts. Nogo KO grafts (right panel) had significantly thicker graft walls compared to those of Nogo WT grafts (left panel). *, lumen. (**B**) Representative H&E image of WT and Nogo KO mouse vein grafts. Scale bar, 100 µm. (**C**) Representative immunofluorescence images of Nogo staining in WT and KO mouse vein grafts; no staining is present in Nogo KO vein grafts. Scale bar, 50 µm. (**D**) Representative image of smooth muscle alpha-actin (SMA) staining of Nogo WT and KO mouse vein grafts. (**E**) Representative images of Nogo WT and KO vein grafts with immunohistochemical staining for F4/80. Arrowheads show vein graft wall. n = 20, 25. (**F**) Line graph shows the time course of vein graft wall thickening as determined by ultrasound imaging. Nogo KO grafts had approximately 40 percent thicker walls compared to those of wild type grafts. *, p = 0.0041; ANOVA. (**G**) Bar graph shows summary of morphological analysis of wall thickness of Nogo WT and KO grafts. *, p = 0.0061; t-test; n = 5. (**H**) Bar graph shows summary of densitometry of F4/80 staining. *, p = 0.0011; t-test. n = 20, 25. (**I**) Bar graphs show summary of proliferation and apoptosis indices in mouse vein grafts. *, p<0.0001, ANOVA; post-hoc testing p<0.05; n = 8, 16, 8, 32 in each of the 4 groups, respectively.

In vivo serial ultrasound imaging showed that vein grafts derived from Nogo KO mice were significantly thicker at days 7, 14 and 21 compared to vein grafts derived from control WT mice (p = 0.0041, n = 28) despite no difference at baseline (day 0) (p = 0.4254, n = 28) (**Figures 3A, 3F**). Increased wall thickness in Nogo KO vein grafts was confirmed using histological analysis (**Figure 3B**); quantification of wall thickness of WT and Nogo KO vein grafts at 21 days showed significant thickening of the Nogo KO intima-media compared to that of the WT vein grafts (**Figure 3G**). Immunofluorescence staining for Nogo-B confirmed prominent and uniform Nogo-B staining within WT vein grafts that was not present in Nogo KO vein grafts (**Figure 3C**). Increased alpha-smooth muscle actin (α-SMA) (**Figure 3D**) as well as increased macrophage F4/80 staining (**Figure 3E**) was observed in Nogo KO vein grafts compared to the amount present in WT vein grafts. Quantification of macrophage F4/80 density within vein grafts confirmed increased density of macrophages in Nogo KO vein grafts (**Figure 3H**). Nogo KO vein grafts demonstrated increased cell turnover compared to WT vein grafts, with increased proliferation and apoptosis, despite no differences at baseline (**Figure 3I**). These data are consistent with a functional role for Nogo-B in vein graft adaptation, and that Nogo-B may limit the number of macrophages present within vein grafts.

### PirB limits macrophage infiltration and vein graft thickening

Since our data suggests that Nogo-B plays a functional role in limiting the number of macrophages present during vein graft adaptation (**Figure 3**), and both Nogo-B and its receptor PirB are present during vein graft adaptation (**Figure 1**), we hypothesized that the mechanism of Nogo-B function in vein grafts is via binding to its receptor PirB on F4/80-positive cells, such as monocytes and macrophages. To test this hypothesis we used our mouse model of vein graft adaptation to compare vein graft adaptation in veins derived from wild type (WT) and PirB KO mice.

**Figure 4:**
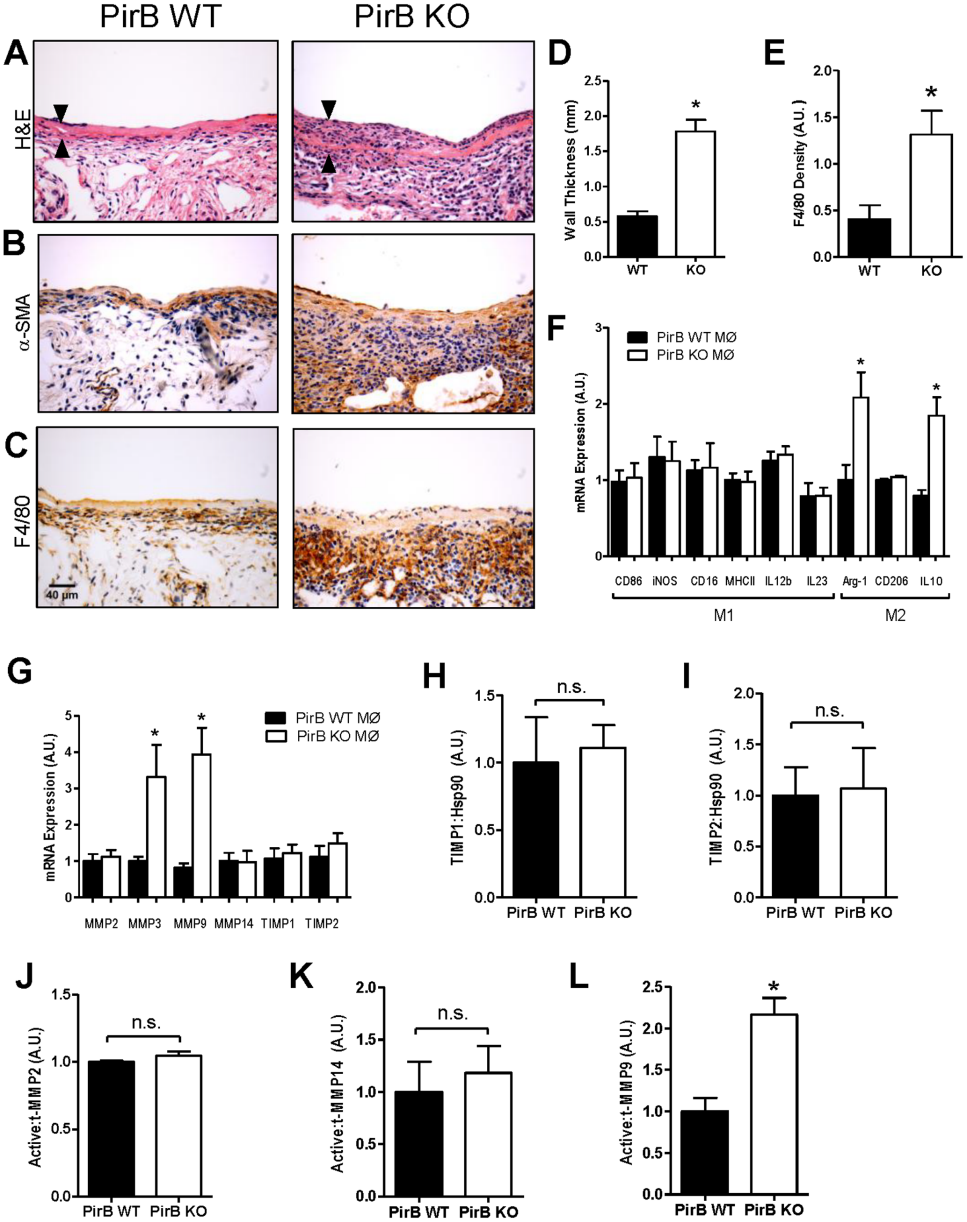
PirB limits F4/80-positive cell infiltration during vein graft adaptation. (**A**) Representative H&E image of PirB WT and KO mouse vein grafts. Scale bar, 100 µm. Arrowheads show vein graft wall. n = 7. (**B**) Representative image of smooth muscle alpha-actin (SMA) staining of PirB WT and KO mouse vein grafts. n = 4. (**C**) Representative images of PirB WT and KO vein grafts with immunohistochemical staining for F4/80. n = 4. (**D**) Bar graph shows summary of morphological analysis of wall thickness of PirB WT and KO grafts. *, p<0.0001, t-test; n = 7. (**E**) Bar graph shows summary of densitometry of F4/80 staining. *, p = 0.0211, t-test; n = 4. (**F**) Bar graph shows summary of mRNA transcript expression in macrophages derived from PirB WT (▪) or PirB KO (□) mice. *, p<0.02, t-test. n = 6. (**G**) Bar graph shows summary of mRNA transcript expression in macrophages derived from PirB WT (▪) or PirB KO (□) mice. *, p<0.03, t-test. n = 6. (**H**) Bar graph shows mean densitometry of TIMP1 immunoreactivity in vein grafts derived from PirB WT (▪) or PirB KO (□) mice. n.s., not significant; p = 0.7859, t-test; n = 3. (**I**) Bar graph shows mean densitometry of TIMP2 immunoreactivity in vein grafts derived from PirB WT (▪) or PirB KO (□) mice. n.s., not significant; p = 0.8941, t-test; n = 3. (**J**) Bar graph shows mean densitometry of MMP2 activity in vein grafts derived from PirB WT (▪) or PirB KO (□) mice. n.s., not significant; p = 0.2252, t-test; n = 3. (**K**) Bar graph shows mean densitometry of MMP14 activity in vein grafts derived from PirB WT (▪) or PirB KO (□) mice. n.s., not significant; p = 0.6569, t-test; n = 4. (**L**) Bar graph shows mean densitometry of MMP9 activity in vein grafts derived from PirB WT (▪) or PirB KO (□) mice. *, p = 0.0109, t-test; n = 3.

Vein grafts derived from PirB KO mice had increased wall thickness compared to vein grafts derived from WT mice (**Figures 4A, 4D**). PirB KO vein grafts showed no difference in lumen area compared to WT vein grafts (6.67±0.36 mm^2^ vs. 6.21±0.45 mm^2^, p = 0.4409, n = 7) but showed increased outward remodeling with increased total vein graft area (8.45±0.40 mm^2^ vs. 6.78±0.41 mm^2^, p = 0.0129, n = 7). Positive α-SMA staining was observed in both PirB WT and KO vein grafts (**Figure 4B**). PirB KO vein grafts demonstrated increased macrophage F4/80 staining within the media and adventitia compared to WT grafts (**Figures 4C, 4E**). Although there were some differences between Nogo KO and PirB KO vein grafts, both vein grafts showed increased intima-media thickening with noticeably increased numbers of macrophages in the vein graft adventitia.

Since these data suggest that binding to PirB on F4/80-positive cells such as monocytes and macrophages may be a mechanism of Nogo-B function during vein graft adaptation, we determined whether macrophages that infiltrate into vein grafts, e.g. macrophages derived from PirB KO mice, have an M1 or M2 phenotype that could be involved in venous remodeling. Although there were no differences in mRNA transcripts of M1 markers between macrophages derived from WT or PirB KO mice, macrophages derived from PirB KO mice had increased numbers of mRNA transcripts of the M2 markers Arginase-1 (Arg1) (n = 0.0188, n = 6) and IL10 (p = 0.0020, n = 6) compared to WT macrophages (**Figure 4F**). These data suggest that PirB may play a role in regulating macrophage M2 phenotype and perhaps venous remodeling.

Since PirB may regulate macrophage M2 phenotype, we determined whether macrophages derived from PirB KO mice have any differences in matrix metalloproteinase (MMP) expression compared to WT macrophages that could be a mechanism for vascular remodeling. Macrophages derived from PirB KO mice showed increased number of mRNA transcripts of MMP3 (p = 0.0278, n = 6) and MMP9 (p = 0.0018, n = 6) compared to macrophages derived from WT mice (**Figure 4G**). There were no differences detected in the number of mRNA transcripts expressed for MMP2, MMP14, TIMP1, and TIMP2 between PirB WT and KO macrophages (**Figure 4G**).

Since macrophages derived from PirB KO mice have increased numbers of M2 markers as well as increased expression of MMP3 and MMP9 compared to macrophages derived from WT mice, we determined whether there were any differences between vein grafts derived from WT or PirB KO mice that reflected these findings. Quantification of densitometry of Western blot analysis of vein grafts derived from WT or PirB KO vein grafts showed no difference in protein levels of TIMP1 (**Figure 4H**), TIMP2 (**Figure 4I**), active-to-total MMP2 levels (**Figure 4J**) and active-to-total MMP14 levels (**Figure 4K**). However, PirB KO vein grafts showed increased active-to-total MMP9 protein compared to WT vein grafts (**Figure 4L**). These data suggest that MMP9 may be a downstream effector of PirB function in F4/80-positive cells such as monocytes or macrophages during vein graft adaptation.

### PirB mediates macrophage-related vascular remodeling

**Figure 5:**
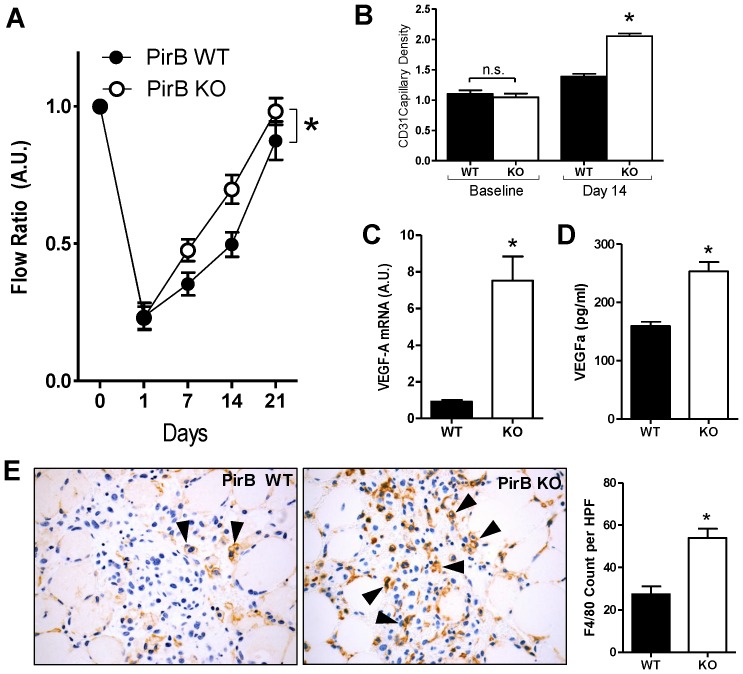
PirB mediates other macrophage-mediated vascular functions. (**A**) Line graph shows mean deep muscle perfusion (ratio ischemic:control leg), 0–21 days after induction of ischemia in PirB WT (▪) or PirB KO (□) mice. *, p<0.0001, ANOVA; n = 10. (**B**) Bar graph show summary of capillary density, at baseline (p = 0.5350, t-test; n = 3) or 14 days after induction of ischemia (*, p<0.0001, t-test; n = 3) in PirB WT (▪) or PirB KO (□) mice. (**C**) Bar graphs show mean VEGF-A mRNA (*, p = 0.001, t-test; n = 5) 72 hours after induction of ischemia in PirB WT (▪) and PirB KO (□) mice. (**D**) Bar graphs show mean VEGF-A protein secretion by PirB WT (▪) and PirB KO macrophages (□) (*, p = 0.0003, t-test; n = 6). (**E**) Representative photomicrographs showing F4/80 staining (black arrowheads) in gastrocnemius muscle 3 days after induction of ischemia in PirB WT (▪) or PirB KO (□) mice. Right panel shows bar graph summarizing mean number of F4/80-positive cells per hpf. *, p = 0.0016, t-test; n = 3.

Since PirB plays a mechanistic role during vein graft adaptation, regulating macrophage function during venous remodeling, we determined whether PirB might play a role in other vascular functions and whether these functions were related to macrophage function. In a hind limb ischemia model, PirB KO mice demonstrated increased flow and faster recovery to baseline compared to WT mice (**Figure 5A**). Histological analysis confirmed the functional increase in the PirB KO response to ischemia, showing increased capillary density in PirB KO mice compared to WT mice despite a similar baseline capillary density (**Figure 5B**). Since increased capillary density in PirB KO mice suggests increased angiogenesis in PirB KO, we examined macrophages derived from PirB WT and KO mice for VEGF-A, a potential stimulus of angiogenesis. Muscle from PirB KO mice showed increased VEGF-A mRNA expression (**Figure 5C**). In vitro PirB KO macrophages showed increased VEGF-A protein secretion compared to macrophages derived from WT mice (**Figure 5D**). To directly test whether the increased remodeling in PirB KO mice in response to hind limb ischemia involved macrophages, the gastrocnemius muscle was examined histologically for F4/80-positive cells; ischemic muscles from PirB KO mice showed increased F4/80 staining in compared to WT mice (**Figure 5E**). These results suggest that PirB mice have increased vascular remodeling in response to hind limb ischemia and that this response is macrophage-dependent.

Since PirB is found on F4/80-positive cells such as monocytes and macrophages and probably not on endothelial cells, as control experiments, two vascular functions that are not macrophage dependent, but are endothelial cell dependent, were also tested. There was no difference in nitric oxide synthesis, either basal or VEGF-A stimulated, between endothelial cells derived from WT or PirB KO mice (data not shown). Similarly, endothelial cells derived from WT or PirB KO mice showed similar amounts of tube formation, both baseline and VEGF-A stimulated (data not shown). These results are consistent with PirB mediating vascular remodeling functions that are F4/80-positive cell-dependent but not wholly endothelial dependent.

## Discussion

We demonstrate that Nogo-B, a mediator of vascular protection, increases in vein grafts during adaptation to the arterial circulation (**Figure 1**); in addition, a receptor for Nogo-B, PirB, increases in coordinate fashion (**Figure 1**) in the vein graft macrophages (**Figures 2**). Loss of Nogo-B is associated with increased vein graft wall thickening and neointimal hyperplasia with a macrophage-predominant inflammatory response (**Figure 3**). Similarly, loss of PirB, an inhibitor of inflammatory cells, also results in increased wall thickening with aberrant perivascular inflammation (**Figure 4**). We also show that macrophages derived from PirB KO mice are associated with an M2 phenotype and may stimulate MMP9-dependent vascular remodeling (**Figure 4**). In addition, PirB may mediate general macrophage-dependent vascular remodeling (**Figure 5**). These results, in toto, suggest that the NogoB-PirB axis helps regulate the inflammatory response that occurs during vein graft adaptation and, more specifically, implicates F4/80-positive cells such as monocytes and macrophages as critical mediators of venous remodeling. As such, vein graft adaptation is not merely a reactive response to injury but rather a necessary constructive adaptation to prevent aberrant neointimal hyperplasia.

Prior work has shown that Nogo-B is prominently expressed within the vascular system and that its loss results in abnormal intimal-medial thickening, implicating a vasculoprotective function. The concurrent finding of Nogo-B loss associated with arterial injury has led to its acceptance as a surrogate marker for vascular injury [2], [14]. Interestingly, this role has largely been derived from arterial models and the decreased Nogo-B expression consistently found following arterial injury [2], [3], [14]. In contrast, Nogo-B and PirB are strongly increased during vein graft adaptation (**Figure 1**). Loss of Nogo-B during vein graft adaptation is associated with increased SMC proliferation and wall thickness (**Figure 3**). These findings are suggestive of aberrant neointimal hyperplasia and infer that the normal role of Nogo-B during vein graft adaptation is to limit the development of aberrant wall thickening. How this function is related to macrophage-dependent remodeling is not clear.

Recently published work has suggested a role for Nogo in the control of macrophage infiltration via interactions of Nogo-B on inflammatory cells and the NgBR on endothelial cells [6]. It is recognized that macrophages play a critical role in vascular remodeling and neointimal hyperplasia that are consistent with our current observations [13], [20]. Macrophage activation remains an important component of vascular remodeling yet there is increasing evidence that suggests neointimal hyperplasia occurs as a result of uncontrolled cellular proliferation and macrophage activity [13], [20], [21]. Within the context of vein graft adaptation, our current work suggests that macrophage inhibition via the Nogo-PirB interaction may be an important mechanism regulating the transition from normal to aberrant vein graft wall thickening. It is not clear, however, whether the Nogo-PirB mechanism affects macrophage influx, egress, and/or function within the vein graft wall. In addition, although PirB may regulate macrophage phenotype and MMP9 expression in vitro (**Figure 4**), the particular subsets of F4/80-positive cells, as well as other PirB-dependent downstream pathways, that play a role during vein graft adaptation in vivo remain poorly defined.

We believe that the importance of this work lies in three inter-related points. First, for decades vascular surgeons have not known whether the macrophage infiltration after vein bypass surgery is beneficial or detrimental, to be stimulated or inhibited; we show that F4/80-positive cells such as monocytes and macrophages are a critical component of vascular remodeling. Second, we show that excessive numbers of macrophages within the vein graft are associated with abnormal wall thickening, such that identifying and targeting F4/80-positive cell activity may be a clinically useful strategy to inhibit vein graft neointimal hyperplasia. Thirdly, we show that vein graft adaptation is not simply a response to injury but also involves adaptive remodeling that is dependent on the inflammatory system. Nogo-PirB control of a subset of inflammatory cells may target a relevant cell type that will be useful for translational therapy.
